# Augmented reality mastectomy surgical planning prototype using the HoloLens template for healthcare technology letters

**DOI:** 10.1049/htl.2019.0091

**Published:** 2019-11-26

**Authors:** Sara Amini, Marta Kersten-Oertel

**Affiliations:** 1 Department of Computer Science and Software Engineering, Concordia University, Montréal, QC, Canada H3G 2V8; 2 PERFORM Centre, Concordia University, Montréal, QC, Canada H4B 1R6

**Keywords:** health care, image reconstruction, augmented reality, medical image processing, surgery, medical computing, prosthetics, augmented reality mastectomy surgical planning prototype, HoloLens template, healthcare technology letters, breast reconstruction, single mastectomy, surgeon, available implants, measurement tools, multiple implants, single patient, augmented reality application, two-chamber implant, holographic implants, traditional way, eliminates sizer implants, system dataset

## Abstract

In breast reconstruction following a single mastectomy, the surgeon needs to choose between tens of available implants to find the one that can reproduce the symmetry of the patient's breasts. However, due to the lack of measurement tools this decision is made purely visually, which means the surgeon has to order multiple implants to confirm the size for every single patient. In this Letter, the authors present an augmented reality application, which enables surgeons to see the shape of the implants, as 3D holograms on the patient's body. They custom developed a two-chamber implant that can gain different shapes and be used to test the system. Furthermore, the system was tested in a user study with 13 subjects. The study showed that subjects were able to do a comparison between real and holographic implants and come to a decision about which should be used. This method can be quicker than the traditional way and eliminates sizer implants from the process. Further advantages of the method include the use of a more accurate, user-friendly device, which is easily extendable as new implants that are on the market can be easily added to the system dataset.

## 1 Introduction

According to the World Health Organisation, breast cancer is the most common cancer in women. It is estimated that over 627,000 women died from breast cancer in 2018 alone [1]. Treatment of breast cancer depends on the stage and type of cancerous tissue and includes chemotherapy treatment, surgery, and/or radiation. One common surgery that is used both to reduce the chance of developing cancer in the future and to remove cancerous tissue is a prophylactic mastectomy. In this type of surgery, one or both breasts are removed completely in order to reduce the risk of cancer reoccurrence or developing breast cancer. Bilateral prophylactic mastectomy (total removal of both breasts) has been shown to reduce the risk of breast cancer for at least 87% for women who have the hereditary breast cancer gene mutation (BRCA1 or BRCA 2) but have yet to be diagnosed with breast cancer, and for 97% for those with a previous diagnosis of the disease [2].

Studies have shown, however, that mastectomy has a negative impact on body image and on the quality of life of women [3]. One way to compensate for the body change caused by mastectomy is to do reconstruction surgery. In breast reconstructive surgery, a prosthesis is used to regain the lost shape and volume of the removed breast(s). The implants, which are usually filled with liquid such as saline or silicone, have a predefined shape and size, and it is up to the surgeon and patient to judge and decide which shape and size are best.

One of the main concerns of patients undergoing reconstructive surgery is the look of the final result. This is especially the case when patients have only one breast removed (single mastectomy); making the modified breast symmetric to the natural one is very important to the patient but can be quite challenging for the surgeon. Dissatisfaction with the result of the surgery can lead to additional surgeries, where the surgeon will try to modify the size and shape of the breast to increase the patient's satisfaction.

The decision about the shape and size of the implant is made by visually comparing available implants with the patient's breast. Currently, there are no tools involved to quantify the accuracy of this choice, and the surgeon has to rely on their experience and ability to predict the final result. As the implant is filled with liquid, it behaves differently when held horizontally or vertically. The shape also changes when the implant is surgically placed inside the patient's body, and the surrounding tissue applies pressure on it. Consequently, the current method that relies on the surgeon's knowledge and experience is prone to human error and not always accurate. Moreover, as the patient does not have the experience of the surgeon, they cannot engage in the process of decision making, as they are unable to imagine the final shape.

In order to compare different shapes, the surgeon has to order a small number of implants for each patient, which is known as ‘sizers’. These implants are then used intraoperatively in the process of decision making, and their only purpose is to give the surgeon visual cues and make it easier for them to choose the right one. After the best match is chosen out of them, the rest are thrown away. As most implants are made out of silicone, they are not biodegradable, and thus the use of sizers can be considered an environmental issue, too.

To mitigate these shortcomings, we present a novel augmented reality (AR) application using the Microsoft HoloLens [4] head-mounted display unit. The application enables surgeons to visualise the final outcome of an implant, without relying on physical implants. In AR, the real world (e.g. a patient in real life) is merged with virtual elements (e.g. virtual breast implants). In our case, we have developed software that allows the surgeon to examine a variety of available breast implant deformations in situ on the patient using AR. By using a marker that is attached to the patient's chest, the final shape can be seen exactly where the implant would be placed, and can easily be compared to the patient's natural breast. This method of visualisation aims to provide the surgeon with improved decision making and surgical planning regarding the implant, thus eliminating the need for sizer implants. It also allows patients to be involved in the process of decision making, as they can have an immersive experience of the final look and provide their opinion. Thus, the main contributions of this study are (i) the development of a first prototype AR system using the Microsoft HoloLens for mastectomy planning, (ii) the evaluation of the ability of users to recognise slight differences in shape of different implant holograms and match them to a physical shape, and (iii) a usability evaluation of the prototype.

The following Letter is organised as follows. Section 2 introduces other academic works that have examined the use of AR for surgery planning, as well as commercial applications of this technology for plastic surgeries. Section 3 describes the design of our software, as well as the custom implant that was developed for the sake of this system. Section 4 presents our experiment, and Section 5 presents the result. In Section 6 we discuss the results and conclusions and future work are given in Section 7.

## 2 Related work

There has been little research aimed at developing AR applications for surgical planning of breast reconstruction surgery. One recent work was proposed by Norberg and Rask [5]. In their work, the Microsoft HoloLens enables users to place a predefined breast shape on a patient's torso and applies a texture similar to a patient's clothing on top of it. This is accomplished by first scanning the patient's body using the HoloLens infrared sensors and smoothing the resulting mesh using a Laplacian filter. The mesh is then used as a base for the breast hologram to appear on. In order to make the hologram more realistic, the texture of patient's shirt is applied to the hologram and saved using the HoloLens web camera. The user can rotate, scale and move the breast hologram to get the desired shape, size, and position. Contrary to our software, their system does not provide shapes acquired by using real implants and is limited to a predefined 3D model, hence the purpose is not to provide computer-assisted decision making in terms of the implant size. This is the only research work we are aware of that focuses on using AR for mastectomy planning.

There are many commercial apps for cosmetic surgery, which let the surgeon modify images of the patient, in order to show them how their body will look like after the surgery. Some of these applications are capable of being used for breast augmentation surgery, such as Mentor's New You Visualizer [6] and Kaeria EURLP's Plastic Surgery Simulator [7]. More complex applications such as Crisalix [8] use a 3D scanned model of a patient's body as their reference and alter the model based on the surgeon's preference. As the results are visualised in 3D, the user has a more realistic understanding of the final look. However, all of the above-mentioned applications show the changes in a model or image, so the user sees the results as an external object and will not see herself in her new body. Illusio [9] has addressed this issue by using marker-based AR. For using their app, the surgeon will wrap the area of interest with a patterned piece of clothing, and then view the patient's body through a tablet camera. The app then substitutes the pattern with the 3D modelled breasts in the process of rendering the video. The surgeon can alter this model in shape, size, and angle, either on each side of both sides. The main difference between the use of software for breast augmentation and breast reconstruction is that the latter must also consider the shape of the natural breast. When only one breast is going to be reconstructed, there is not much that can be done to change the size and shape of the natural breast. Hence, the most important aim of our software is to compare available implants with the patient's breast and suggest the one that is the most symmetric with the breast that is going to be preserved.

AR has also been used in other surgeries preoperatively. Fushima and Kobayashi [10] suggested a mixed reality system to be used in maxillofacial surgery. They use the results of 3D-computed tomography to reconstruct the 3D shape of the patient's skull and jaw bones. This model is synchronised with a dental cast using three titanium spheres as reference points. Pre-operative planning is done by transforming and moving the 3D cast model. In comparison with the work above, our work does not rely on any medical images, and thus requires different solutions.

## 3 System description

**Fig. 1:**
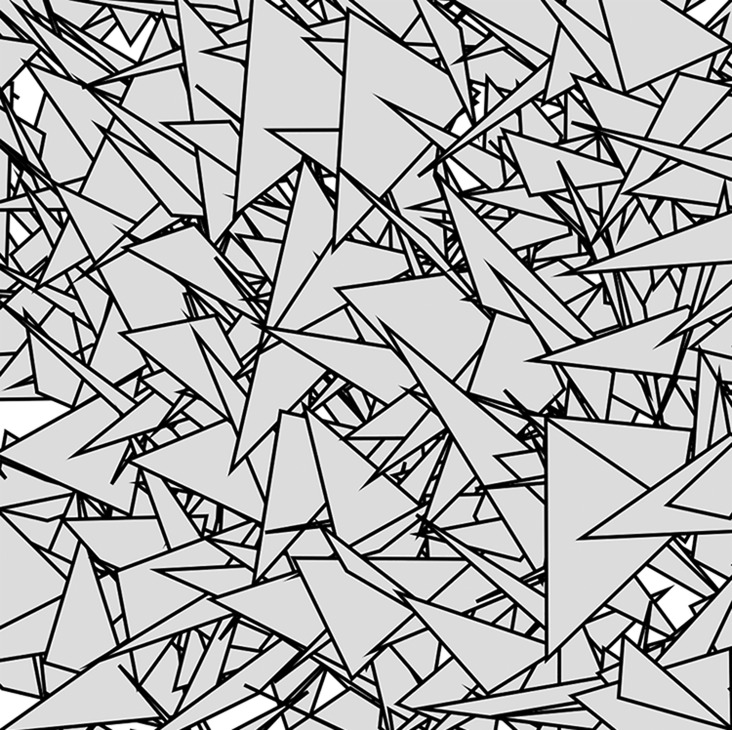
Pattern used as a marker for our AR application which uses Vuforia. An ideal marker for use with Vuforia has to be rich in detail, have good contrast, and not include any repeating pattern. This texture was generated by covering a plain surface with random triangle outlines

Our system comprises a HoloLens device running in development mode, a custom developed AR application, and a predefined pattern used as the marker for AR (see Fig. 1). The application was built using Unity version 2018.1.0 and uses the Vuforia SDK [11] to handle the AR functionality. Vuforia allows developers to define patterns as markers that can then be used for marker-based AR. A database of these patterns is then created and the markers can then be downloaded and added to a given application. An ideal marker for use with Vuforia has to be rich in detail, have good contrast, and should not include any repeating pattern. In order to satisfy these conditions, a texture was generated by covering a plain surface with random triangle outlines (Fig. 1).

**Fig. 2:**
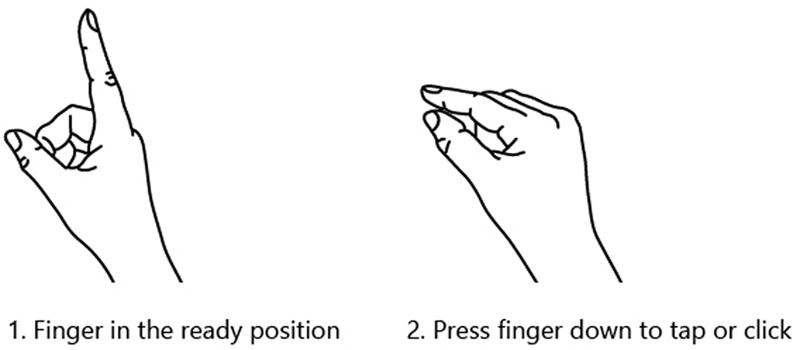
Air tap hand gesture [12] used for selecting the menu items in our application

Vuforia also has a feature called extended tracking, which saves the position of the marker in the world; using this feature, even if the marker is lost from the user's point of view, the augmented object will keep its last known position. The developed application runs on the HoloLens device, which both serves as the camera and handles user inputs (hand gestures and gaze movement). The user can interact with the application using a holographic user interface (UI). The UI consists of a pointer that can be moved by head rotation, and three holographic buttons that can be selected by gazing at them and triggered by doing a hand gesture. We used air tap as trigger, which is one of the default gestures that HoloLens is capable of recognising, and is performed by the user holding their hand upright, raising their index finger (ready state) pressing their finger down (tap) and back up (release state), as can be seen in Fig. 2. The UI follows the user's head rotation and position, and it is always visible in the field of view of the user. The buttons allow the user to choose between seven different implant shapes (described below), and to hide the menu.

It should be noted that voice recognition was also tested in an early version of the prototype; however, we chose the air tap as voice inputs were often not recognised by the system, particularly when users had an accent when speaking the English commands.

**Fig. 3:**
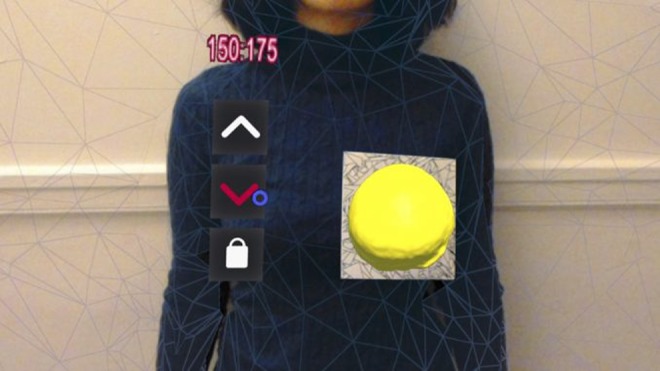
Screenshot of the HoloLens application during use. The marker is attached to the patient's clothing, and the hologram of implant is placed on the centre of the marker. The Holographic menu can be seen on left, as well as the cursor which is placed on ‘down’ button. The amount of saline needed to be injected in each chamber to gain this shape can also be seen on top of the menu, in millilitres

The application starts with the HoloLens scanning the environment using its video camera and looking for the marker we have defined in our Vuforia database. Since the implant model is set to appear on top of the marker, the position of the marker is quite important. The marker, which is 17.5 cm × 17.5 cm, must be attached to the patient's clothing or body, where the implant will be placed. When the marker is found, the implant hologram will be placed there. After the HoloLens detects the marker, the first available shape appears as a hologram in the middle of the marker by default, assuming that the marker is in the correct position. Using the extended tracking feature of Vuforia, we ensure the user can walk around the patient and look at the shape from different angles, without losing sight of the implant when the tracker is out of the point of view of the HoloLens. The user can also use the up and down buttons by gazing at them and performing an air tap, in order to navigate between available shapes and find the best match. Each button changes colour when the user gazes at it, hence the user knows when the pointer is where they want to click (Fig. 3). Moreover, the cursor changes to a hand shape when the user's hand is detected in the field of view of the HoloLens in a ready state, so they know when the device is enabled to react to their hand gesture.

**Fig. 4:**
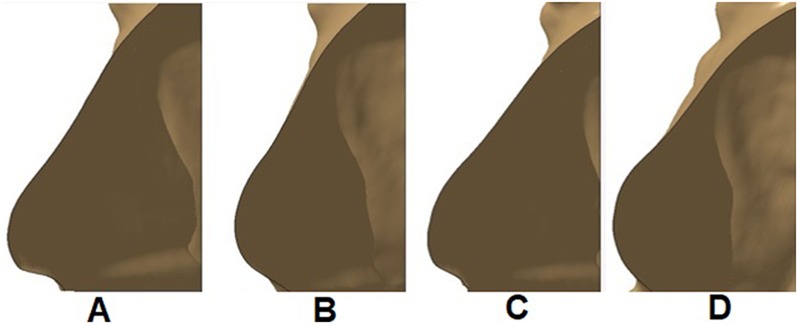
Application features six different shapes based on a custom implant that we developed with two chambers. Four of the custom shapes gained by using our developed implant shown on a medical mannequin are depicted here

The application features six different shapes based on a custom implant that we developed with two chambers. The two chambers, which can be filled with water using separate input tubes, enabled us to create a variety of different and more natural shapes. This implant was cast by pouring Dragon Skin silicone [13] type 35, in a 3D printed mould that was created using CATIA software [14]. The varying shapes were gained by injecting different volumes of water in each chamber (Table 1) and attaching the water-filled implant to a medical mannequin wearing a special stretch shirt. The shirt mimics the role of skin by adding pressure on the implant and deforming it (see Fig. 4).

**Table 1:** Volume of water injected in the custom implant for each shape

Shapes	Volume added to the upper chamber, ml	Volume added to the lower chamber, ml
A	150	235
B	150	175
C	210	175
D	210	140
E	245	200
F	245	150
G	105	105

After injecting the desired volume of water into each chamber, the K-Scan software [15], which allows for scanning objects in 3D by using a photogrammetry technique, was used to scan the resulting 3D shape. We used a Microsoft Xbox One Kinect sensor [16], which has a depth camera, in order to take multiple scans from the implant from different angles. These scans, which consist of a dense point cloud of the scene, were inserted into K-Scan, aligned and merged together, and created a 3D model of the scene. We then cropped the implant shape out of this model and saved it as a .obj file. The application has access to these files in the memory and can summon them based on the user's input.

## 4 Experiment

**Fig. 5:**
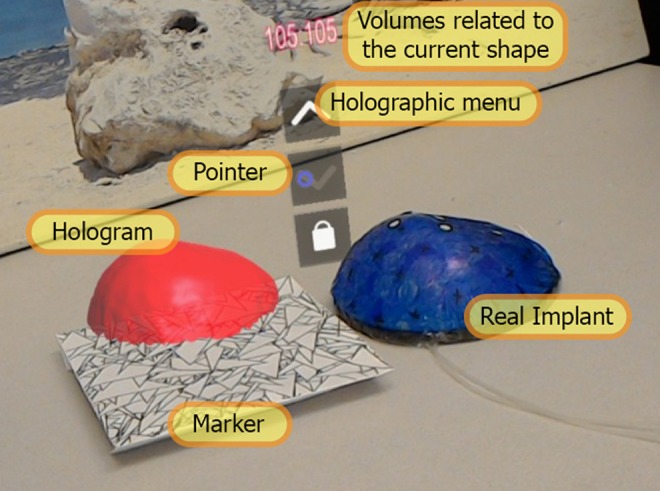
Experimental set-up used in our study required subjects to match the holographic 3D shapes available in the application with a real implant that was filled with 110 ml of saline in each of its chambers. The implant is covered with elastic wrapping to mimic the setup which was used for scanning shapes in Table 1

We tested the usability of our software in a user study, where participants were asked to browse the seven available implant shapes and compare them to a real implant. The implant used in this experiment was the same custom implant that was used to create the shapes mentioned in Table 1, and it was inflated with 110 ml of water in the top chamber and 110 ml in the lower chamber to create shape G with some slight change in the size. The real implant was placed on a flat surface and covered with an elastic covering to mimic the environment that was created in the scanning process (see Fig. 5). The marker was also put on the same surface next to the implant, so the holographic implant will appear side by side to the real implant, which serves the role of the patient's natural breast in this concept. The marker position was chosen based on users' personal preferences. In addition to the cursor and the buttons giving feedback to the users' inputs, the 3D models were assigned different colours to let the user know when they have successfully triggered a button to observe the next model.

Our study sample was composed of 13 subjects (aged 21–40 (median 26), five females and eight males). They were all students, studying computer science, software engineering, electrical engineering or mechanical engineering. Eight of the participants had previous experience with the HoloLens or had seen how HoloLens works before. Nevertheless, all subjects were briefed on the concept of AR and holograms, HoloLens functionality, and the reason for the study. They also received a briefing on the system functionality, i.e. the HoloLens input system (hand gestures and gaze movements) and how to interact with the UI. Before the beginning of the trials, the subjects were presented with the test environment and were asked to interact with the UI to summon different shapes on the marker. The goal of this pre-test session was to let subjects practice in order to reduce potential learning bias.

The subjects were informed that there might be no exact match to the real implant, and they need to find the shape that looks the most similar. No time limit was set for the decision-making process, and the subjects were supervised by the researcher to guide them about the system when necessary. After the subjects completed the study, they were asked to fill out the system usability scale (SUS) [17] questionnaire, which is a standard usability questionnaire. SUS consists of ten five-point questions and is a well-known test that is used to estimate the usability of a system. A system that scores 68 or above is considered usable and above average. The subjects were also asked to rank the system on a scale of 1–5, on how helpful it was in comparing shapes and finding the best match.

## 5 Results

On average, it took the subjects 7 min and 28 s to choose the best matching shape and declare it. Each subject took from 2 min and 44 s to 20 min and 47 s, with a median of 6 min and 11 s. The time taken to see all the shapes and compare them to the real implant for the first time varied from 2 min and 21 s to 9 min and 48 s, with an average of 3 min and 43 s. As this time was close to all subjects, the variation between times that took each subject to make a decision seems to be related to subjects' personal sense of making sure of their final choice. In terms of subjects' choice of shape, as can be seen in Table 2, seven subjects chose shape G as the best match which was considered to be the correct answer. Other shapes that were suggested by the subjects include shapes A, E, F, being chosen 3, 1, and 2 times, respectively. There was no need to move the marker during the process of decision making, and the setting remained unchanged for each trial.

**Table 2:** Shapes that were chosen as the best match

Shape	Times being chosen
G	7
A	3
E	1
F	2

In terms of qualitative evaluation, the SUS score given by the users varies from 97.5 to 50.5 (see Table 3) with a median of 70. The system was considered to be well integrated, with 57 positive points out of the possible 65 points. However, 10 out of 13 users found the system cumbersome to use, eight believed that a technical person must always be present to support the users in order for them to be able to use the system correctly, and 12 believed that the system is unnecessarily complex. The cumulative scores given for these topics were 33, 36, and 26, respectively.

**Table 3:** Usability assessment done using SUS. The subjects that are distinguished with a star sign had previous experience with HoloLens

SUS question/subject	S1*	S2*	S3*	S4*	S5*	S6*	S7	S8	S9*	S10	S11	S12*	S13	Sum
I think that I would like to use this system frequently	4	3	2	5	5	2	4	3	2	3	3	4	2	42
I found the system unnecessarily complex	1	1	1	1	2	2	3	3	2	1	4	3	2	26
I thought the system was easy to use	5	5	5	3	4	4	5	3	4	2	2	3	3	48
I think that I would need the support of a technical person to be able to use this system	1	2	2	1	2	3	3	1	4	4	4	4	5	36
I found the various functions in this system were well integrated	5	5	5	5	5	5	3	4	5	3	5	4	3	57
I thought there was too much inconsistency in this system	1	1	1	1	1	2	1	2	2	2	1	3	1	19
I would imagine that most people would learn to use this system very quickly	5	2	4	3	5	5	4	4	4	5	4	4	5	54
I found the system very cumbersome to use	1	1	1	3	2	2	3	3	3	2	4	4	4	33
I felt very confident using the system	5	5	5	4	3	4	4	3	4	4	5	3	3	52
I needed to learn a lot of things before I could get going with this system	1	1	2	1	2	1	2	1	3	3	3	3	4	27
SUS score	97.5	85	85	82.5	82.5	75	70	67.5	62.5	62.5	57.5	52.5	50	—

Overall, the system gained an average of 71.5 on the SUS scale, meaning that it passed in this evaluation by 3.5 points. Out of 13 subjects, 12 users found the software useful for comparing shapes and objects, giving it an average score of 4.23 out of 5.

## 6 Discussion

It can be deduced from Table 3 that the users’ previous knowledge about the HoloLens affects their experience with this system. On average, the users who knew about or had tried HoloLens before had an easier time learning how to interact with the UI and gave better scores to the system. These users are marked with a star next to their ID number in Table 3. However, both groups equally believe that the presence of someone as technical support would be necessary to have a successful experience with this application.

The main problem stated by the users was detecting the position of the cursor when the application starts. The HoloLens needed to be recalibrated for every single user to make sure they can see the cursor, the menu, and the holograms. Otherwise, the cursor could not be seen in the middle of the frame. This process takes 5–10 min and makes the whole experience more time consuming and tiring. Furthermore, as the cursor is controlled by the user's head, it is not as accurate as users expect it to be. The cursor tends to move with every slight body movement and seems to be constantly shaking with users' breathing.

Another issue was caused by the limited field of view of HoloLens, which subjects needed to remember in order to let it detect the hand gestures. Although the cursor provides feedback when the user's hand is detected in ready state (Fig. 2) and turns into a hand shape, all subjects had to be reminded, constantly, to do the hand gestures in a way that the HoloLens can detect their hand movement. Moreover, being forced to stretch their arms in front of their body to fulfil this goal, was tiresome for the subjects and one of the main downfalls of the system. The subjects also found it difficult to get used to controlling the cursor by gaze movement, as they naturally do not turn their heads to look at an object where they can see it just by moving their eyes over it. The users did not believe they would use an AR system in the long-term frequently. However, all users believed that most people can learn how to work with this system quickly, with this question gaining 54 points out of 65 possible points.

It is also important to note that all subjects had an engineering or computer science background, and none was a plastic surgeon or had experience in decision-making and mastectomy planning. Two aspects are important to note, first, it is possible that having an engineering background made it easier for them to understand technical terms and made their learning phase shorter. Second, all of the participants lacked the experience that plastic surgeons have from years of matching implants with human breasts. Hence, it can be hypothesised that once a surgeon passes the learning phase and learns how to interact with the HoloLens device, it would take them less time (in comparison with ordinary users) to choose the best matching shape out of the 3D models. In future work, we will study the impact of the system on plastic surgeons.

Another main distinction between our study and clinical practice is the number of available implants in real life, which makes the process of decision making more complex. For this study, as we did not have access to cosmetic implants or a patient, a custom-made implant was used both for creating 3D models instead of a cosmetic implant and for being compared against these shapes instead of the patient's real breast.

Unlike Nornberg and Rask's work [5], the 3D models in our system are coloured with plain colours and do not use the texture from the patient's body. One main reason for this shortcoming is the presence of the marker, which is used to detect the breast area but on the other hand blocks visual access to patient's skin or shirt in that area. One possible solution is to access the skin tone or the shirt texture by taking a picture from the patient beforehand. However, for the sake of this study it was not an issue, as the implant itself was being compared to the 3D models. This feature will be added in future work.

The current system relies on the use of a third-party platform for detecting the marker and placing the 3D model at the marker location. In the next steps of this work that will involve patients, we can eliminate the need for the marker and the third-party platform by automatically detecting patient's breasts by using visual features of their body.

We scored 71.5 for SUS, which makes our system slightly above the threshold to be considered usable. Since many of the issues that were raised involved interactions with HoloLens, it can be assumed that the score can be increased by using a simpler device. One possible approach would be to use mobile devices such as the iPad, which have a more tangible interaction interface and one which users are more familiar with.

## 7 Conclusion and future work

Our preliminary study showed that the idea of using AR to find similarities in objects is considered useful by the users. Users were able to compare 3D models with a real object, without the need of having any of the reference objects present. Moreover, as all 3D models appear on the same spot on the marker, once the marker was on the right spot in relation to the implant, there was no need to set the position for each model, which saves time and makes the comparison less error prone by eliminating the effect of the change of implant position.

However, the HoloLens was shown not to be the best platform to implement this system. All of the main problems stated by the users are related to the HoloLens and how to interact with them. Since the HoloLens behaves very differently from other devices that the users interact with on a daily basis, they have to go through a learning phase to be ready to use the system. Moreover, the way user input is received by the HoloLens, such as stretching an arm to do a hand gesture, was considered tiresome for the users and undesired. These obstacles make the system not an ideal option to be used on a long-term basis as all users stated. The system passed the SUS evaluation by 3.5 points, and it appears that resolving these issues could improve the usability of the system even more. Usability can further be improved by shifting to a platform that the users are more familiar with, such as mobile devices. Using devices that include a touch screen can help with cursor accuracy, eliminates calibration time, and is less tiresome for users to keep using for a long time. Moreover, in order for this system to be useful for surgeons, the database of the 3D models must feature shapes related to real implants that are currently used for reconstruction.

Although this work was done on the basis of a need analysis with plastic surgeons, our study did not involve domain experts. In the next part of this work, we will work with domain experts to determine how the use of the system could help in decision making and reduction of the use of sizers during surgery.

As mentioned in Section 1, one of the main challenges for surgeons is determining the deformation of an implant in different positions. We did not address this issue in this simplified implementation of the application, the current system can only show the shape of the implant in one position. However, if the system were able to predict the behaviour of the implant in different positions, it would have a huge advantage over current methods. In the future we will look at finite element method modelling to determine the deformation of the implant and natural breast in different positions.
